# Hepatitis C Virus Infection: Molecular Pathways to Insulin resistance

**DOI:** 10.1186/1743-422X-8-474

**Published:** 2011-10-18

**Authors:** Fahed Parvaiz, Sobia Manzoor, Huma Tariq, Farakh Javed, Kaneez Fatima, Ishtiaq Qadri

**Affiliations:** 1 NUST Center of Virology and Immunology (NCVI), National University of Sciences and Technology (NUST), Islamabad 44000, Pakistan

**Keywords:** HCV Infection, Molecular Pathways, Insulin Resistance

## Abstract

Chronic Hepatitis C virus has the potential of inducing insulin resistance and type 2 Diabetes Mellitus *in vitro *as well as *in vivo *. Structural and non-structural proteins of HCV modulate cellular gene expression in such a way that insulin signaling is hampered, concomitantly leads toward diabetes mellitus. A number of mechanisms have been proposed in regard to the HCV induced insulin resistance involving the upregulation of Inflammatory cytokine TNF-α, hypophosphorylation of IRS-1 and IRS-2, phosphorylation of Akt, up-regulation of gluconeogenic genes, accumulation of lipids and targeting lipid storage organelles. This review provides an insight of molecular mechanisms by which HCV structural and non-structural proteins can induce insulin resistance.

## Background

HCV, a blood born pathogen, belongs to the family Flavivridae, infects hepatocytes and was discovered in 1989. The genome size of HCV is 9.6 Kb that encodes about 3010 amino acids and gets translated into structural and non-structural proteins [1-3].

HCV is a major cause of acute and chronic liver disease worldwide [4-6]. Acute HCV infection becomes persistent in about 85% of cases [7]. Chronic infection with HCV is a multifaceted disorder associated with insulin resistance, glomerulonephritis, B-cell lymphoma and type 2 diabetes mellitus [8]. It is estimated that 3.3% of the population globally (lower in Europe 1.03% and highest in Africa 5.3%) and 10% of the Pakistani population is chronically infected with HCV [9-12]. Until now, there is no vaccine against HCV that can provide effective immunization [13].

The process of glucose uptake is quite complicated that involves binding of insulin to the insulin receptor and subsequently binds to Insulin receptor substrate 1 (IRS-1), activating a number of different kinds of proteins like PIK3, PDC and PIP3 which in turns, activates GLUT4 and causes the translocation of glucose from exoplasmic surface to the inside of cell [14]. After glucose is internalized, glucose gets phosphorylated by hexokinase, enters glycolysis and gets converted into pyruvate. This glucose is taken up by the adipocytes and is utilized in the formation of lipids while, in case of muscles, glucose is converted into glycogen and glycogenolysis is inhibited by insulin. In liver, insulin downregulates glucose level by inhibiting gluconeogensis and glycogenolysis involving Phosphoenolpyruvate carboxy kinase (PEPCK), key regulator of gluconeogensis [15,16].

Any change in this signaling is likely to induce insulin resistance which is associated with a number of pathophysiological changes including glucose intolerance, obesity, dyslipidemia and hypertension. During the course of insulin resistance several inflammatory cytokines and lipid metabolites like free fatty acids interrupt with the normal insulin signaling and promote type 2 diabetes mellitus [14].

## HCV Induced Insulin resistance

Insulin resistance is a pre-diabetic phase that is frequently observed in chronic HCV patients (25%) and lesser in case of other hepatic disorders including hepatitis B virus (10%) [17]. Non-alcoholic fatty liver disease (NAFLD) is one of the most important causes of chronic infections and Insulin resistance (IR) represents the hallmark of NAFLD. It is a systemic disorder that not only infects liver but also nervous system, pancreas, heart, kidney and muscles [18]. IR is a condition where adequate amount of insulin is required to maintain the glucose level or normal insulin concentration is unable to maintain homeostasis [19,20]. Glucose intolerance is related to diminish sustained virological response that promotes insulin resistance, a step leading towards steatosis and hepatocellular carcinoma [21,22]. Although the characteristics and complications of HCV are well identified, but the molecular mechanisms of HCV induced IR and hepatocellular carcinoma are yet to be fully understood [23,24].

Hepatitis C virus infects the hepatocytes, progresses through chronic phase and eventually leads towards IR, Type 2 Diabetes mellitus and Steatosis. The prevalence of Type 2 diabetes mellitus in chronic HCV patient ranges from 24-50% and this frequency is about 5 times greater than the rest of Hepatic cirrhosis [25,26].

Several studies revealed that HCV infection promotes the release of tumor necrosis factor alpha (TNF-α). These metabolic disorders like IR and steatosis are dependent on the sustained virological response (SVR) i.e. reduction of IR and steatosis will be favored in response to the high SVR [27].

## Role of HCV structural and non-structural proteins in Insulin Resistance

**Figure 1:**
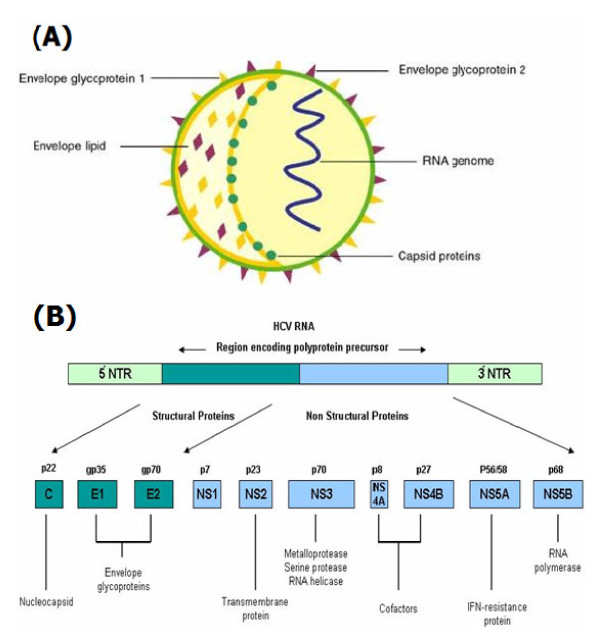
**Model Structure of HCV**: Section A: Generalized structure of Hepatitis C virus, Section B: Genomic organization of HCV that gets translated into three structural and seven non-structural proteins

The genome size of HCV is about 9.6 kb which gets translated into 10 different structural and non structural proteins. These proteins help virus to replicate and damage host machinery. However HCV proteins Core, NS-3 and NS-5 are mainly involved in IR. The core protein is involved in the formation of capsid, NS-3 contains helicase and proteolytic activity, NS-5A downregulates interferon stimulated genes and NS-5B is a RNA polymerase [28,29]. The structural organization of HCV genome is given the figure. A brief role of HCV structural and non-structural proteins in the induction of insulin resistance is discussed here (Figure 1).

### Core Protein

HCV Core protein is a pathogenic feature of this virus that can induce several metabolic disorders in the host cell. HCV induces insulin resistance by knocking out PPAR 28□ by the way of core protein induction. PPAR 28□-/- core Ag leads to the hypophosphorylation of Insulin receptor substrate (IRS)-1 as well as 2 and phosphorylation of Akt [30]. Another study has showed similar results in which HCV core transgenic Huh 7 and HepG2 cells expressed low levels of IRS-1 and IRS-2 with the concomitant increased expression of SOCS-3 [31]. HCV Core transgenic mice were shown to block P85 subunit of PI3K, a downstream insulin signaling molecule, and impair insulin signaling pathways [32].

TNF-α, antioxidant gene, is found to be released in an excess and is required for the phosphorylation of serine residues of IRS-1 eventually leading to the downregulation of downstream insulin signaling molecule Akt. HCV core protein increases the expression level of TNF-α and promotes insulin resistance [33]. In response to this oxidative stress, four major DNA glycosylases are unregulated i.e. NTH1, OGG1, NEIL1 and NEIL2. HCV Structural as well as Non-Structural proteins interferes with these glycosylases. Expression of NEIL1 is strongly down regulated by Core protein and to a lesser extent by NS-3, NS-4A and NS-5A [34].

### NS-3 Protein

HCV Non structural protein 3 (NS-3) has been shown to induce oxidative stress by the way of reactive oxygen species (ROS). During viral replication, NS-3 induces the upregulation of Nicotinamide adenine dinucleotide phosphate oxidase 2 (NOX2) which, in turns, accelerates the production of ROS eventually leading to the modulation in downstream signaling pathways like hepatic fibrosis [35]. It has been found that NS-3 cans downregulate T-cells and natural killer cells thereby promoting its proteolytic activity [36]. Secondary structure of NS-3 has the potential of inducing neoplastic transformation and can lead towards carcinoma [37]. Yet its direct role in the induction of insulin resistance has not been determined.

### NS-5A Protein

NS5A potentially interacts with RNA dependant kinase (PKR), linked to the IFN stimulated genes, downregulates PKR and consequently diminishes the IFN response against HCV RNA [28]. There is correlation between the interferon responsiveness and the phosphorylation of eIF-2α. NS5A expression masks the effect of PKR which, in turns, lowers the phosphorylation of eIF-2α and promotes interferon resistance [38].

HCV Non Structural protein 5A (NS-5A) co-localizes on the ER membrane, promotes lipid accumulation and reactive oxygen species (ROS) that modulate intracellular signaling involving transcriptional factors like NF-kB, STAT3 and Ca^2+ ^ions and promote damage to the hepatocytes [39]. By this way of damage to the hepatocytes, NS5A induces ER stress which leads to insulin resistance directly or indirectly by the upregulation of cellular gene protein phosphatase 2A (PP2A) [40,41]. PP2A has been shown to downregulate Akt, which in turns, hampers the insulin signaling as well as it induces interferon resistance. Therefore, NS5A plays a pivotal role in the developing interferon resistance and takes its path towards IR [42,43].

Studies on transgenic mice expressing NS-5A reveals that this protein interacts with Apolipoprotein-1 of the fatty hepatocytes, hampers the lipid transport, accumulates cholesteryl esters and thereby leads towards liver pathology including Steatosis and hepatocellular carcinoma [44].

**Figure 2:**
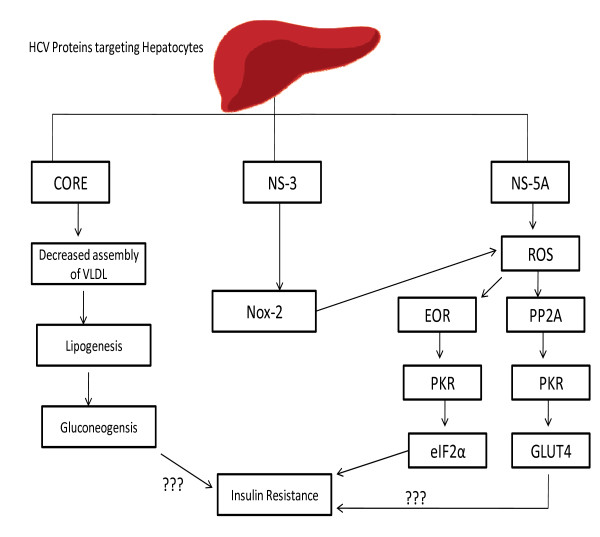
**Schematic Representation of HCV Induced Insulin Resistance**: Figure demonstrates that HCV non-structural proteins (NS-3 and NS-5A) and structural protein (Core Protein) modulate various cellular genes that are involved in insulin resistance.

HCV NS-5A has great potential of interacting with ER and inducing stress on the ER homeostasis by the way of upregulating ER over load Response (EOR). During this phenomenon, one of the key transcriptional factors of untranslated protein response ATF6 is upregulated. However, the exact mechanism by which NS5A is inducing the EOR is still remaining unknown [38]. This Figure illustrates the sequential pathways involving HCV induced insulin resistance (Figure 2).

## Role of cellular factors contributing in HCV Induced Insulin Resistance

Some of the cellular factors which are found to be involved in promoting insulin resistance are explained here.

### Role of cytokines

Various HCV proteins interact with the endoplasmic reticulum and mitochondria induces oxidative stress with the concomitant upregulation of TNF-α and some other cytokines like Interleukin8, Interleukin6, Tumor growth factor-β and Fas ligand [45]. As TNF-α is critically important for the determination of HCV infection. TNF-α is an inflammatory cytokine that is strongly upregulated in HCV infection and downregulates the insulin signaling mechanisms by blocking the phosphorylation of key molecules Insulin receptor Substrates (IRS) and hampers the GLUT4 translocation for the glucose molecule across the plasma membrane of the host cell. [46].

### Role of IRS

Depending upon the genotype, HCV induced Insulin resistance can be promoted by a number of mechanisms like Genotype 1 can deteriorate IRS-1 by the ubiquitinylation favored by SOCS-3. In case of genotype 1b, IRS-1 is down regulated by mTOR (mammalian target of Rapamycin) that causes the serine/threonine phosphorylation of IRS-1 and hampers normal insulin signaling. In genotype 3, there is enhanced production of SOCS7 that diminishes IRS-1 and promotes insulin resistance [47-49].

### Role of Akt

Akt, the downstream insulin signaling molecule, effectively governs the transport of glucose through a controlled feedback mechanism involving PKC and protein tyrosine phosphatase 1B (PTP1B). Insulin signaling is favored by doing the phosphorylation of PTP-1B. As a negative feedback mechanism, PKCζ can phosphorylate the serine residues of IRS-1 thereby blocking the IRS-1/PI3K complex and inhibit insulin signaling [50]. IRS can activate a number of insulin linked proteins like Src2 homology domain containing proteins, Fyn, CSK, CRK, NCK as well as Akt. However, Akt is given more importance because of its strong role in insulin signaling. As far as IRS dependant Akt mediated signaling is concerned, it activates PDK which, in turns, phophorylates and activates two proteins Akt (also known as PKB) and PKC with the dominant isoforms zeta and lambda. Although Akt predominates in this pathway, still a number of other cellular factors have to be explored that are crucially involved in this pathway [51]. Recent evidence shows that Huh 8 cells expressing HCV NS-5A induced IR by the way of downregulating serine-473residue of Akt and Insulin receptor-β [52].

### Role of lipogenic genes

Previously it was thought that insulin resistance is just because of the excessive glucose or imbalanced glucose metabolism. However, it is now revealed that insulin resistance is strongly influenced by abnormalities in the lipid metabolism. Any dysfunction of the leptin triggers lipotoxicity through the production of free fatty acids in skeletal muscles, myocardium and pancreatic islets thereby promoting insulin resistance [53]. After the HCV infection, core protein down-regulates microsomal triglyceride transfer protein (MTP), an enzyme that mediates lipids translocation to the ER membrane and decreases the assembly of very low density lipoproteins (VLDL) [54]. Consequently, there is an enhanced production of lipids that promotes insulin dependent steatosis in a MAP kinase pathway [55]. AMP-activated protein kinase (AMPK) is involved in controlling lipid as well as glucose metabolism. During the HCV replication, AMPK is phosphorylated at threonine 172, promoting lipid accumulation thereby favoring HCV induced insulin resistance [56].

### Role of gluconeogenic genes

HCV infection promotes the expression of gluconeogenic genes which, in turns, enhances insulin resistance. It has been observed that HCV promotes fatty acid synthesis by the upregulation of lipogenic gene sterol regulatory element binding protein 1c which promotes the transcriptional activation of other lipogenic genes like acetyl CoA carboxylase, ATP citrate lyase, hydroxymethylglutaryl CoA reductase etc [57].

Findings of a recent study have revealed that in HCV infected cell line Huh.8, PEPCK which is a key regulator of gluconeogenesis, as well as cellular lipids was strongly upregulated under HCV NS-5A expression. This is indicating a plausible role of NS-5A in gluconeogenesis and imbalances in the glucose metabolism [45].

## Conclusion

HCV is a multifaceted disorder that involves different cellular and viral factors for the disease progression. Chronic HCV infection is more likely to favor IR by the way of HCV core, NS-3 and NS-5A protein. These proteins are under strict investigation because there analogues can prove to be effective treatment against HCV infection. We can conclude that HCV induced IR is not merely because of glucose imbalances rather it involves upregulation of the gluconeogenic and lipogenic genes that promote glucose intolerance and progresses towards IR, a step towards hepatocellular carcinoma.

## Abbreviations

HCV: Hepatitis C virus; IRS: insulin receptor substrate; GLUT: glucose transporter; PEPCK: phosphoenol pyruvate carboxykinase; NAFLD: non-alcoholic fatty liver disease; IR: insulin resistance; TNF-α: tumor necrosis factor alpha; SVR: sustained virological response; NS: non-structural proteins; ROS: reactive oxygen species; NOX2: nicotinamide adenine dinucleotide phosphate oxidase 2; PKR: protein kinase R; eIF: eukaryotic initiation factor; ER: endoplasmic reticulum; STAT: signal transducer and activator of transcription; PP2A: protein phosphatase 2A; ATF: activated transcription factor; MAP: mitogen activated pathway, AMPK: AMP-activated protein kinase;

## Competing interests

The authors declare that they have no competing interests.

## Authors' contributions

FP and SM reviewed the literature, and wrote the manuscript. IQ edited the manuscript. HT, FJ and KF helped FP and SM in literature review. All the authors read and approved the final manuscript.
